# Nociceptin Increases Antioxidant Expression in the Kidney, Liver and Brain of Diabetic Rats

**DOI:** 10.3390/biology10070621

**Published:** 2021-07-03

**Authors:** Ernest Adeghate, Crystal M. D’Souza, Zulqarnain Saeed, Saeeda Al Jaberi, Saeed Tariq, Huba Kalász, Kornélia Tekes, Ernest A. Adeghate

**Affiliations:** 1 Department of Pharmacodynamics, Semmelweis University, Nagyvárad Tér 4, H-1089 Budapest, Hungary; Ernest14@gmail.com (E.A.); drtekes@gmail.com (K.T.); 2 Department of Anatomy, College of Medicine & Health Sciences, United Arab Emirates University, Al Ain P.O. Box 17666, United Arab Emirates; crystal.dz@uaeu.ac.ae (C.M.D.); 201250300@uaeu.ac.ae (S.A.J.); stariq@uaeu.ac.ae (S.T.); 3 Department of Psychology and Social Work, Flinders University, Bedford Park 5042, Australia; saee0034@flinders.edu.au; 4 Department of Pharmacology and Pharmacotherapy, Faculty of Medicine, Semmelweis University, H-1089 Budapest, Hungary; drkalasz@gmail.com; 5 Zayed Foundation, United Arab Emirates University, Al Ain P.O. Box 17666, United Arab Emirates

**Keywords:** nociceptin, kidney, liver, brain, hippocampus, neuropeptides, cFOS, antioxidants immunohistochemistry

## Abstract

**Simple Summary:**

Nociceptin (NC) is a small peptide implicated in the physiology of pain, learning and memory. Here we investigated the role of NC in the induction of antioxidants in the kidney, liver, and the brain of diabetic rats using morphological and biochemical methods. Normal and diabetic animals were treated with NC for 5 days. Catalase (CAT) was expressed in the kidney, liver, and the neurons of the brain. Although CAT was markedly (*p* < 0.05) lower in the tubules of the kidney of normal and diabetic animals after NC treatment, NC significantly (*p* < 0.001) increased the presence of CAT in the liver and brain of diabetic rats. Superoxide dismutase (SOD) was observed in kidney tubules, hepatocytes, and neurons of the brain. Treatment with NC markedly (*p* < 0.001) increased the level of SOD in hepatocytes and neurons of the brain. Glutathione reductase (GRED) was seen in the convoluted tubules of the kidney, hepatocytes and neurons of the brain. Treatment with NC markedly increased (*p* < 0.001) the expression of GRED in kidney tubules, hepatocytes and neurons of the brain. In conclusion, NC can help diabetic patients mitigate the effects of oxidative stress by its ability to induce endogenous antioxidants.

**Abstract:**

Nociceptin (NC) consists of 17 amino acids (aa) and takes part in the processing of learning and memory. The role of NC in the induction of endogenous antioxidants in still unclear. We examined the effect of NC on the expression of endogenous antioxidants in kidney, liver, cerebral cortex (CC), and hippocampus after the onset of diabetes mellitus, using enzyme-linked immunosorbent assay and immunohistochemistry. Exogenous NC (aa chain 1–17; 10 µg/kg body weight) was given intraperitoneally to normal and diabetic rats for 5 days. Our results showed that catalase (CAT) is present in the proximal (PCT) and distal (DCT) convoluted tubules of kidney, hepatocytes, and neurons of CC and hippocampus. The expression of CAT was significantly (*p* < 0.05) reduced in the kidney of normal and diabetic rats after treatment with NC. However, NC markedly (*p* < 0.001) increased the expression CAT in the liver and neurons of CC of diabetic rats. Superoxide dismutase (SOD) is widely distributed in the PCT and DCT of kidney, hepatocytes, and neurons of CC and hippocampus. NC significantly (*p* < 0.001) increased the expression of SOD in hepatocytes and neurons of CC and the hippocampus but not in the kidney. Glutathione reductase (GRED) was observed in kidney tubules, hepatocytes and neurons of the brain. NC markedly increased (*p* < 0.001) the expression of GRED in PCT and DCT cells of the kidney and hepatocytes of liver and neurons of CC. In conclusion, NC is a strong inducer of CAT, SOD, and GRED expression in the kidney, liver and brain of diabetic rats.

## 1. Introduction

### 1.1. Oxidative Stress

Oxidative stress occurs in biological systems when the tissue pool of free radicals exceeds the capability of cellular antioxidants to neutralize them. Oxidative stress is described as a disparity between pro-oxidants and anti-oxidants with a concomitant disturbance in the whole redox network and a subsequent destruction of macromolecules [1]. Free radicals, such as hydrogen peroxide (H_2_O_2_), superoxide (O_2_•−), hydroxyl (•OH), and singlet oxygen (^1^O_2_) are produced during the course of cell metabolism [2,3] or other biological processes in the body. Free radicals are released from intrinsic sources such as mitochondria peroxisomes or during inflammation, ischemia, and exercise [4,5]. These free radicals can also be released extrinsically during the smoking of tobacco, exposure to pollutants of the environment, radiation, heavy metals, drugs, and many other noxious agents [4,6].

These free radicals cause biochemical, as well as structural, defects in cells membranes because they induce peroxidation of lipids, including those located in the plasma membranes of almost all cells, in addition to the induction of breaks in DNA strands. Structural damage to the membrane will impair the receptors and other key molecules embedded within the plasma membrane. All of these will affect the normal function of the cell because of malondialdehyde induced conformational changes in receptors, enzymes, and other key transduction molecules. Indeed, it has been shown that lipid peroxidation is a catalyst for the development of various diseases including, but not limited to, a large number of pathological conditions, such as inflammation and neurodegeneration [7].

In contrast to the noxious and deleterious effects of free radicals, free radicals have been shown to have important and vital roles on normal cell function. For example, macrophages and other phagocytes produce, store, and release free radicals to destroy pathogenic microorganisms that invade the body. However, these free radicals have to be present in optimal concentrations conducive to the normal functioning of the body system [8]. Moreover, it is well known that nitric oxide is an important neurotransmitter in the nervous system, at least when present in suitable non-toxic levels [9,10,11]. Studies from our laboratory have also shown that NO enhances insulin release from the pancreas [12,13]. Neurons of the enteric nervous system contain nitric oxide synthase, an enzyme that metabolizes NO [14]. NO is also a key molecule in the regulation of blood flow in the vascular bed. This effect is initiated by vasodilation [15].

### 1.2. Nociceptin, Oxidative Stress and Diabetes Mellitus

Since oxidative stress is a major cause of cell, tissue, and organ dysfunction, and eventually organ failure, attempts have been made to target bioactive molecules involved in the neutralization of free radicals. Studies using supplements of enzymatic endogenous antioxidants such as SOD, CAT, and glutathione peroxidase to treat diseases have been reported [16,17]. Many studies have also been performed looking into the ability of non-enzymatic antioxidants (vitamin C, E, tocopherol) to either prevent and/or cure a large variety of diseases, including diabetes mellitus [18,19]. These reports showed that vitamin C and E have beneficial effects on the outcome of diseases [18,19,20,21,22,23]. The addition of hyperglycemia increases the degree of oxidative stress in the body because increased glucose level is a major trigger for the release of free radicals [24]. However, it is worth noting that clinical trials of antioxidants in human subjects have not provided definitive curative roles of these antioxidants [25].

There is a strong search, by researchers, to find new bioactive agents to reduce and mitigate the impact of free radicals, especially in diabetes mellitus, where oxidative stress is a major cause of debilitating morbidity and mortality because they induce the release of free radicals [24,26]. Proteins and peptides can act as antioxidants because they have the capacity to prevent lipid peroxidation and neutralization of free radicals [27]. As a peptide, nociceptin, and its analogues, have been tested for their ability to neutralize the damage caused to neuroblastoma cells by H_2_O_2_ [28]. The outcome has been controversial and far from conclusive, and the localization of endogenous antioxidants was not attempted. The effect of nociceptin on the antioxidant activity in the liver and the kidney has not been reported. Increased nociceptin gene expression has been reported in neuroglia cells in response to oxidative stress [28], indicating that nociceptin may indeed be implicated in the regulation of oxidative stress. Other studies showed that the cerebrospinal fluid (CSF) level of nociceptin increased significantly with a concomitant increase in the level of CSF of superoxide [29,30]. Several studies [31,32] have also indicated that the administration of nociceptin protect the gastric mucosa. Studies in our laboratory showed that nociceptin is present in pancreatic beta cells [33], where they are capable of stimulating insulin release [34]. Insulin has been reported to be a powerful antioxidant against diabetes-induced oxidative stress [35]. Based on these reports, we hypothesized that nociceptin can increase the tissue expression of endogenous antioxidants to exert its beneficial effects.

The aim of the study, therefore, is to determine whether exogenous administration of nociceptin will have any acute impact on the tissue expression of selected endogenous antioxidants (catalase, superoxide dismutase, glutathione reductase) in the kidney, liver, and brain of rats after the onset of diabetes mellitus.

## 2. Materials and Methods

### 2.1. Experimental Animals

Wistar rats, weighing 250–300 g, were used for the experiment. Although the experimental animals were bred at the Animal House Facility, College of Medicine & Health Sciences, United Arab Emirates University, Al Ain, UAE, the stock of the Wistar rats was acquired from Harlan Laboratories (Oxon, England, UK). The experimental rats were maintained in groups of four rats per plastic cage at a temperature of 23 °C with a cycle of 12 h day and 12 h night. The animals had water and chow (Emirates Feed Factory, Abu Dhabi, United Arab Emirates) ad libitum.

### 2.2. Diabetes Mellitus Induction

Wistar rats were made diabetic using a single dose of 60 mg/kg^−1^ body weight of streptozotocin (STZ; Sigma, Poole, UK). STZ was given intraperitoneally after dissolving it in a citrate acid buffer solution, as described in previous experiments [36]. The same volume of the buffer solution was given intraperitoneally to normal and diabetic control rats. One Touch II Glucometer (Life Scan Inc., Johnson and Johnson, Chesterbrook, PA, USA) was used to determine whether the rats were diabetic. Rats with glucose level ≥ 126 mg/dL were chosen for the study. All rats were humanely euthanized 5 days after treatment with nociceptin. The study was performed based on the guidelines of the Declaration of Helsinki, and approved by College of Medicine & Health Sciences Animal Research Ethics Committee (#A5-14).

### 2.3. Experimental Groups

Rats (*n* = 24) were assigned, at random, into four different groups: I. Normal control (*n* = 6): Normal male Wistar rats treated with 10 µg/kg body wt. of physiological saline. II. Normal treated (*n* = 6): Normal male Wistar rats treated with 10 µg/kg body wt. of nociceptin (amino acid chain 1–17). III. Diabetic control (*n* = 6): Diabetic male Wistar rats treated with 10 µg/kg body wt. of physiological saline. IV. Diabetic treated (*n* = 6): Diabetic male Wistar rats treated with 10 µg/kg body wt. of nociceptin (amino acid chain 1–17). The nociceptin dose administered in this study is similar to those used in previous experiments [37].

### 2.4. In Vivo Treatment of Rats with Nociceptin

Nociceptin (abcam ab38198; aa1-17), dissolved in phosphate buffered saline was administered intraperitoneally at 10 µg/kg daily for 5 days to control as well as diabetic rats. Nociceptin was given at 9:00 am every day. Equal quantity of phosphate buffered saline (vehicle) was given intraperitoneally to another set of 6 normal rats (normal control), diabetic rats (diabetic controls) at the same time and for the same time period. Nociceptin was administered 2 weeks after the onset of diabetes mellitus, leaving enough time for STZ to be completely eliminated from the body. This short duration of the treatment was meant to examine the acute effects of nociceptin.

### 2.5. Tissue Collection, Fixation and Paraffin Embedding

At the end of the experiment, the rats were anaesthetized with ether, and kidney, liver, and brain (at the level of the hippocampus) tissues were expeditiously removed, trimmed of connective tissues, and cut into 3–4 mm^3^ pieces. The tissue samples were then fixed in Zamboni’s solution for 3 days. Following tissue fixation, the samples were embedded in paraffin wax according to a previously described technique [38]. Briefly, kidney, liver, and brain tissue samples were dehydrated in ascending concentrations of ethanol (50% to 100%), cleared in xylene, followed by embedding in liquid paraffin wax at 55 °C.

### 2.6. Immunofluorescence Study of Endogenous Antioxidants

Six µm thick sections were made using with Shandon AS325 microtome (Kalamazoo, MI, USA), treated in a hot bath to soften the tissue. The tissue sections were later mounted on glass slides coated with gelatin to facilitate adhesion. The glass-mounted slides were then kept on a hot plate for 2 h. The tissue sections were processed for double labeled immunofluorescence, according to a previously reported procedure [38]. In brief, paraffin was removed from the tissue sections using xylene and hydrated with a sequence of graded ethyl alcohol before eventual treatment with citrate buffered solution. The tissue sections were incubated for 24 h with the primary antibody followed by an overnight incubation in the secondary antibody (Table 1). Incubation in the primary antibodies were performed at 4 °C. The sections were incubated in immune conjugated TRITC after treatment with primary antibodies. The sections were then washed in PBS and mounted with cover slides with CITI-Fluore media (Science Services GmbH, München, Germany). The images were captured with an AxioCam HRc digital camera using AxioVision 3.0 Software (Carl Zeiss, Oberkochen, Germany). Images were processed using Image J 1.8.

### 2.7. Immunolocalization of Neural Nitric Oxide Synthase and cFOS Protein

Staining of the C3 hippocampal region was conducted to show whether intraperitoneal administration of nociceptin for 5 days could induce the expression of cFos protein, an indication of increased activity of neurons.

### 2.8. Primary and Secondary Antibodies

The list of the primary and secondary antibodies and their dilutions is given in Table 1. Superoxide dismutase, Anti-Catalase, Anti-Glutathione Reductase from Abcam (Cambridge, MA, USA), while FITC, 1:1000 and TRITC were bought from Jackson ImmunoResearch Laboratories (Ely, Cambridgeshire, UK).

### 2.9. Measurements of Catalase Activity

Catalase activity was measured in normal, normal treated, diabetic untreated, and diabetic treated with nociceptin, using the colorimetric method with commercial kits (Cayman Chemical, Ann Arbor, MI, USA). The ethical permit to run catalase activity was (#A5-14) issued by the College of Medicine & Health Sciences Animal Research Ethics Committee, UAEU, Al Ain, United Arab Emirates.

### 2.10. Densitometric Analysis of Immunofluorescence

The density of the immunofluorescence staining of catalase (CAT), superoxide dismutase (SOD), and glutathione reductase (GRED) containing structure were measured using Image J software^®^ (NIH, Bethesda, MA, USA). Briefly, the image was copied to the clipboard and inserted on 8-bit slot of Image J. The image was then inverted, and the line tool was then used to gather the total number of pixels. The pixel peaks were then analyzed as percentages of control. The process was done for 6 different sections per group, and the data analyzed as mean ± SEM. The control (normal) image was considered as 100%. Images were analyzed in the kidney from the renal cortex, in the liver from the area in the immediate vicinity of central veins, and the area between layers II and VI in the cerebral cortex. Image analysis for the hippocampus was done at the central part of the CA3 region.

### 2.11. Statistical Analysis

All experimental data were calculated as mean ± standard error of the mean. Differences between the groups were calculated using One-way ANOVA. Significant differences between mean values of the group, and two different timelines, were calculated with an unpaired *t*-test. Statistical significance was set at a value of *p* < 0.05.

## 3. Results

### 3.1. Nociceptin and Endogenous Antioxidants in Kidney

The characteristics of the animals used in the study is provided in Table S1. 

**Figure 1:**
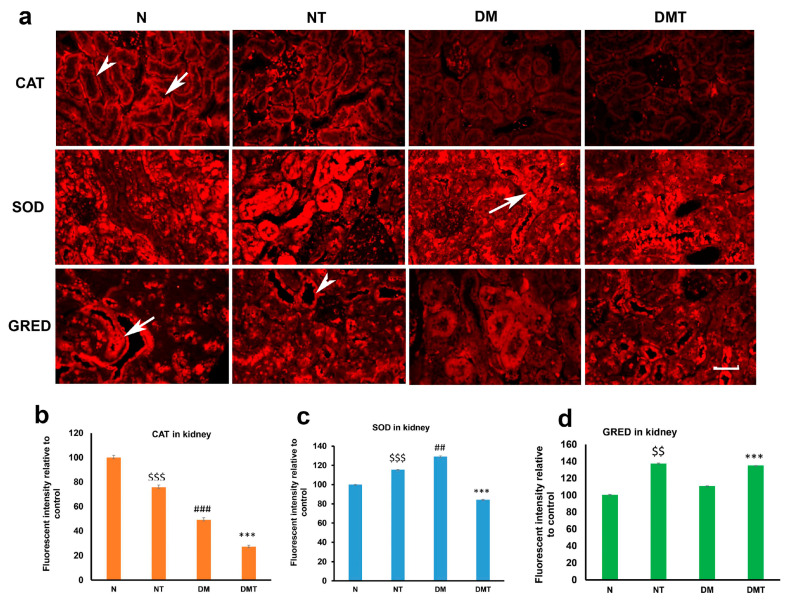
Images (**a**) of immunohistochemistry showing catalase (CAT), superoxide dismutase (SOD), and glutathione reductase (GRED) expression in the cortex of the kidney of normal (N), normal treated (NT), diabetic untreated (DM) and diabetic treated with nociceptin (DMT). While nociceptin has variable effects on CAT and SOD expression, the tissue expression of GRED increased significantly after nociceptin treatment in both normal and diabetic rats (**b**–**d**). Proximal convoluted tubules (arrow); distal convoluted tubules (arrow head); *n* = 6; Scale bar = 25 µm; Magnification = ×400; $$ and $$$ (normal treated versus normal untreated); ## and ### (diabetic untreated versus normal untreated); *** (diabetic untreated versus diabetic treated) $$ *p* < 0.05, $$$ *p* < 0.001; *** *p* < 0.001; ### *p*< 0.001.

**Figure 2:**
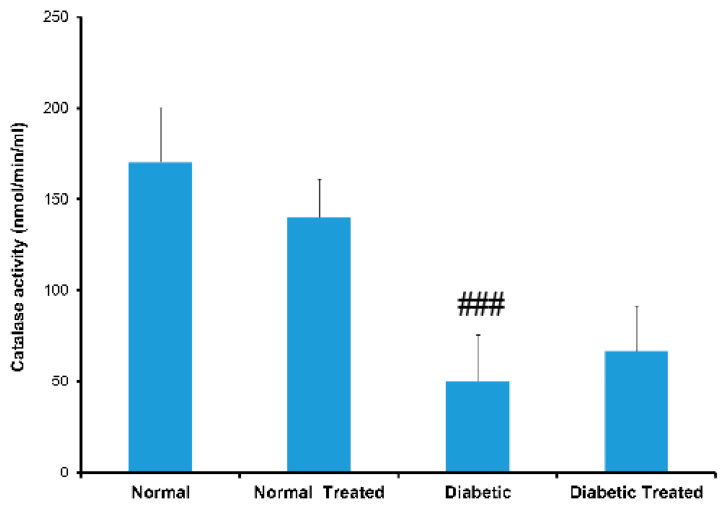
Histograms of showing catalase (CAT) activity in the serum of normal (N), normal treated (NT), diabetic untreated (DM) and diabetic treated with nociceptin (DMT). Catalase activity was significantly reduced in untreated diabetic rats compared to normal. Note that there was no significant changes in CAT activity after treatment with nociceptin. *n* = 6; ### (diabetic untreated versus normal untreated); *p* < 0.01.

Catalase (CAT) was present in the proximal (PCT) and distal (DCT) convoluted tubules of normal and diabetic rats. However, the intensity of CAT was significantly reduced after nociceptin treatment in normal and diabetic rats compared to untreated controls (Figure 1a,b and Figure 2). The expression of superoxide dismutase (SOD), on the other hand, was elevated in normal rats treated with nociceptin. In contrast, the expression of SOD was markedly reduced in diabetic rats treated with nociceptin (Figure 1a,c). In a way that was completely different from CAT and SOD, glutathione reductase (GRED) expression in kidney tissues was significantly increased in normal, as well as diabetic, rats treated with nociceptin (Figure 1a,d).

### 3.2. Nociceptin and Endogenous Antioxidants in Liver

**Figure 3:**
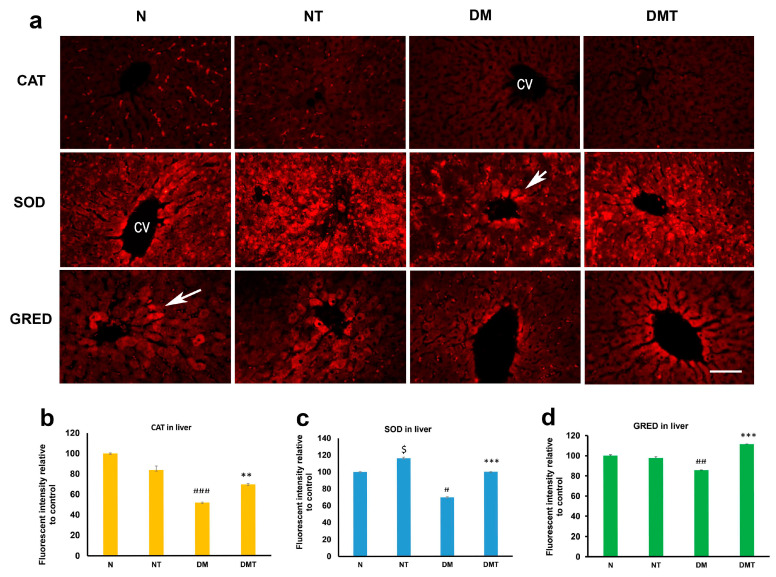
Fluorescence images (**a**) of catalase (CAT), superoxide dismutase (SOD), and glutathione reductase (GRED) expression in the liver of normal (N), normal treated (NT), diabetic untreated (DM), and diabetic treated with nociceptin (DMT). Note that the expression of SOD was significantly higher in normal and diabetic rats treated with nociceptin. Nociceptin also markedly increased the tissue level of GRED after nociceptin treatment in diabetic rats (**b**–**d**). cv = central vein; *n* = 6; Scale bar = 25 µm; Magnification = ×400; $ (normal treated versus normal untreated); ## and ### (diabetic untreated versus normal untreated); ** and *** (diabetic untreated versus diabetic treated). $ *p* < 0.05; # *p* < 0.05; ## *p* < 0.01; ### *p* < 0.001; ** *p* < 0.05; *** *p* < 0.001.

Catalase (CAT) was discernible in hepatocytes located around the central vein of the liver. CAT expression was markedly lower in the liver of rats treated with nociceptin when compared to untreated controls (Figure 3a,b). In contrast, superoxide dismutase (SOD) was strongly expressed in liver cells located in the immediate vicinity of central veins. The expression of SOD was significantly elevated in both normal and diabetic rats treated with nociceptin, compared to untreated rats (Figure 3a,c). Moreover, nociceptin was able to increase the tissue level of glutathione reductase (GRED) in hepatocytes of diabetic rats when compared to untreated controls (Figure 3a,d).

### 3.3. Nociceptin and Endogenous Antioxidants in the Cerebral Cortex

**Figure 4:**
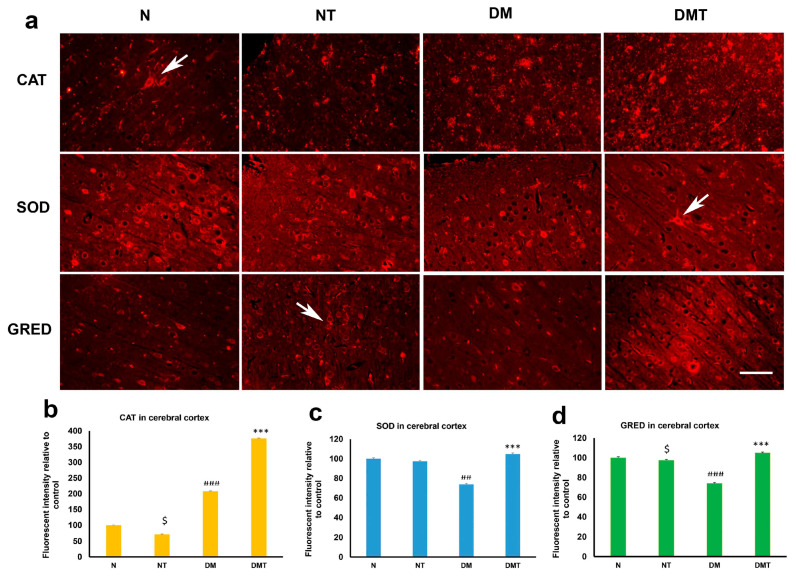
Immunofluorescence images (**a**) of catalase (CAT), superoxide dismutase (SOD), and glutathione reductase (GRED) expression in the cerebral cortex of normal (N), normal treated (NT), diabetic untreated (DM) and diabetic treated with nociceptin (DMT). Note that the expressions of CAT, SOD and GRED were significantly higher in neurons (arrows) of cerebral cortex of diabetic rats treated with nociceptin when compared to controls (**b–d**). *n* = 6, Scale bar = 25 µm; Magnification = ×400; $ (normal treated versus normal untreated); ## and ### (diabetic untreated versus normal untreated); *** (diabetic untreated versus diabetic treated) $ *p* < 0.05; ## *p* < 0.01; ### *p* < 0.001; *** *p* < 0.001.

Neuronal cell bodies contain catalase (CAT). While nociceptin induced a lower expression of CAT in normal tissue, it caused a large, and significant, elevation in the tissue level of CAT in the cerebral cortex of diabetic rats compared to untreated controls (Figure 4a,b). Nociceptin did not have any effect on the expression of superoxide dismutase (SOD) in the cerebral cortex of normal rats. However, it significantly elevated the SOD level in the neurons of the cerebral cortex of diabetic rats. The expression of SOD was significantly reduced in the cerebral cortex of untreated diabetic rats compared to treated and untreated normal rats (Figure 4a,c). Nociceptin reduced the expression of glutathione reductase (GRED) in the neurons of the cerebral cortex of normal rats but increased GRED level in the cortex of diabetic rats when compared to untreated controls (Figure 4a,d, Figures S1–S3).

### 3.4. Nociceptin and Endogenous Antioxidants in Cornu Ammonis 3 (CA3) Region of Hippocampus

**Figure 5:**
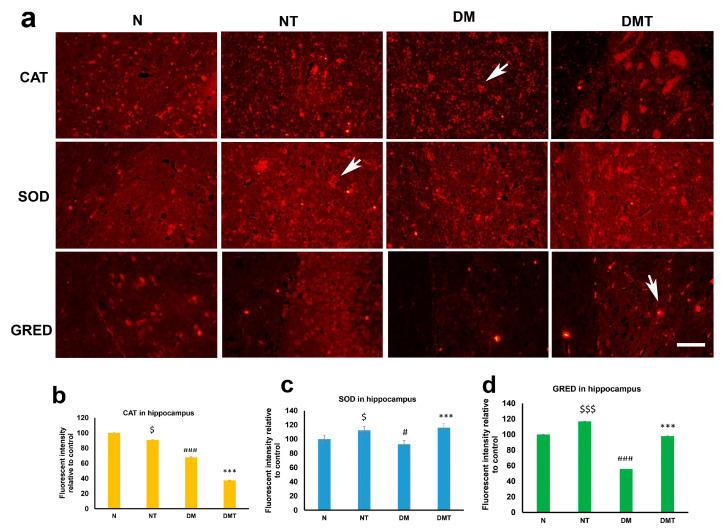
Immunofluorescence images (**a**) of catalase (CAT), superoxide dismutase (SOD), and glutathione reductase (GRED) expression in the CA3 region of the hippocampus of normal (N), normal treated (NT), diabetic untreated (DM) and diabetic treated with nociceptin (DMT). Note that the expressions of CAT is reduced after nociceptin treatment. However, nociceptin caused large and significant increases in SOD and GRED expression in the neurons (arrows) of hippocampus and normal and diabetic rats compared to controls (**b**–**d**). *n* = 6, Scale bar = 25 µm; Magnification = ×400; $ and $$$ (normal treated versus normal untreated); # and ### (diabetic untreated versus normal untreated); *** (diabetic untreated versus diabetic treated) $ *p* < 0.05; $$$ *p* < 0.01; ### *p* < 0.001; *** *p* < 0.001.

Immunofluorescence staining shows that endogenous antioxidants are present in neurons of the CA3 region of the hippocampus. Nociceptin caused a reduced expression of CAT in the CA3 region of the hippocampus in both normal and diabetic rats. In contrast to its inability to induce CAT expression in the hippocampus of normal and diabetic rats, nociceptin enhanced large increases in SOD expression in the hippocampus of both normal and diabetic rats, when compared to untreated controls (Figure 5a,b). Nociceptin also actuated an increase in the level of expression of superoxide dismutase (SOD) in the hippocampus of both normal and diabetic rats (Figure 5a,c). In a similar trend, nociceptin caused a large and significant increment in the expression of glutathione reductase (GRED) in the neurons of the hippocampus of both normal and diabetic rats (Figure 5a,d).

### 3.5. Neuronal Nitric Oxide Synthase (nNOS) and cFOS in CA3 Region of Hippocampus

The hippocampus of normal, normal treated, diabetic untreated, and diabetic rats treated with nociceptin were processed for nNOS and cFOS to determine whether nociceptin has any effect on nNOS and cFOS. nNOS has been shown to play an important role in the production of nitric oxide from L-arginine substrate and in the development of the nervous system. It also plays a role in the maintenance of learning and memory circuits in the hippocampus [39]. In addition, previous studies have reported that increase in the expression of cFOS, in neuronal nuclei, is indicative of neuronal activity and a recent depolarization [40]. Our aim in this part of the study was to determine whether nociceptin has any role in the initiation of any of these events.

**Figure 6:**
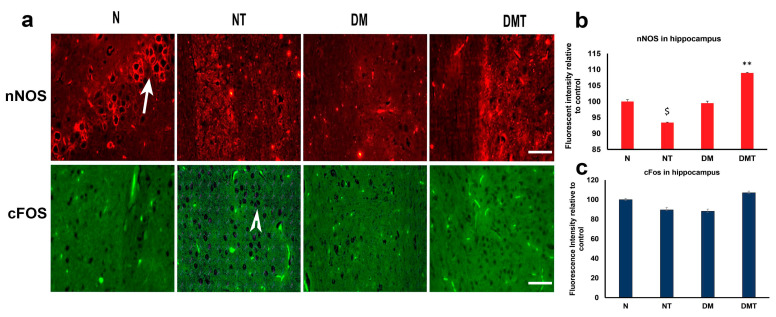
Immunofluorescence images (**a**) of nitric oxide synthase (nNOS) and c-FOS expression in the CA3 region of the hippocampus of normal (N), normal treated (NT), diabetic untreated (DM), and diabetic treated with nociceptin (DMT). Note that the expressions of nNOS is reduced after nociceptin treatment in normal but upregulated in diabetic rats treated with nociceptin. In contrast, nociceptin failed to stimulate cFOS expression (arrow head) in the hippocampus of either normal or diabetic rats compared to controls (**b**,**c**). *n* = 6, Scale bar = 25 µm; Magnification = ×400; $ (normal treated versus normal untreated); ** (diabetic untreated versus diabetic treated) $ *p* < 0.05; ** *p* < 0.01.

Nociceptin induced a marked reduction in the expression of nNOS in CA3 neurons of normal rats compared to controls. In contrast, nociceptin caused a significant increase in nNOS level in the neurons of the CA3 region of the hippocampus of diabetic rats (Figure 6a,b).

Regarding cFos, nociceptin failed to induce the activation of this biomarker of neuronal activation in normal and diabetic rats (Figure 6a,c).

## 4. Discussion

### 4.1. Nociceptin and Endogenous Antioxidants in Kidney

Catalase (CAT) is ubiquitous to many cells and organs. It helps to neutralize H_2_O_2_, released during metabolic activities, into harmless oxygen and water [41]. The human variant of CAT consists of four subunits capable of neutralizing millions of H_2_O_2_ molecules [42]. We showed that CAT is present in the proximal, as well as distal, convoluted tubules of the kidney of normal rats. This observation agrees with that of Johkura et al. [43], who demonstrated the presence of CAT in developing kidneys, using immunohistochemistry. Apart from this, most study examined the plasma level of CAT rather than its tissue level in the kidney. We hereby show that CAT is present in the proximal (PCT), as well as the distal convoluted, tubules (DCT) of the kidney of normal and diabetic rats. The expression of CAT was lower in an untreated diabetic kidney compared to untreated controls. The degree of CAT expression was significantly lower in nociceptin-treated normal rat kidney compared to untreated normal controls. Superoxide dismutase (SOD) reduces ROS in tissues and has a strong anti-inflammatory effect [44]. Three types of SOD exist, SOD1 is found mainly in the cytoplasm, SOD2 is in the mitochondria, and SOD3, which is located in the extracellular space [45]. It catalyzes superoxide ions into oxygen and H_2_O_2_, and the H_2_O_2_ is eventually neutralized by catalase [46]. The SOD used in this study contains all of the isoforms and its expression is highly elevated in the kidney of normal rats treated with nociceptin. In contrast, the expression of SOD was significantly lower in diabetic rat kidneys treated with nociceptin. The reason behind this is unknown, but it may be due to the fact that the SOD present in the kidney has been exhausted in diabetic rat kidneys. Previous studies have shown that the structure of the kidney is grossly impaired after the onset of diabetes [47]. The third endogenous antioxidant examined was glutathione reductase (GRED). GRED reduces GSSG to GSH, which helps to convert H_2_O_2_ to harmless H_2_O [48]. The administration of nociceptin significantly increased the expression of GRED in kidney tissues in normal and diabetic rats. This shows that nociceptin has the capacity to increase the level of a subset of endogenous antioxidants in not just normal but also in diabetic rats.

### 4.2. Nociceptin and Endogenous Antioxidants in Liver

The expression of catalase (CAT) was significantly lower in the liver of untreated diabetic rats when compared to that of normal untreated rat. This observation was confirmed by enzyme-linked immunosorbent assay (ELISA). Previous reports have indeed shown decreased level of CAT after the onset of diabetes mellitus [35]. The level of CAT expression increased after treatment with nociceptin. ELISA technique showed that the blood level of CAT rose, but not significantly, after treatment of diabetic rats with nociceptin. The authors are not aware of any other study that reported the effect of nociceptin on catalase expression in the liver.

Here we showed that superoxide dismutase (SOD) is widely distributed in the parenchyma of liver of normal and diabetic rats. However, the expression of SOD is significantly enhanced in both normal and diabetic rats after nociceptin administration. The expression of SOD was mostly localized to hepatocytes located in the vicinity of and around the central veins. The reason for this pattern of expression is not clear. It is possible that the SOD, located in this area of the liver, may be destined to neutralize free radicals resident in the blood around this part of the liver. To the best of our knowledge, this is the first report on the ability of nociceptin to significantly stimulate the expression of SOD in the liver.

The results of this study showed that glutathione reductase (GRED) was widely observed in liver cells around the central veins. The expression of GRED was significantly enhanced in hepatocytes around the central veins after nociceptin treatment, especially in diabetic rats. This observation shows that most of the endogenous antioxidants, including SOD, may indeed be concentrated within hepatocytes located around the central veins, where they are most probably needed.

### 4.3. Nociceptin and Endogenous Antioxidants in the Cerebral Cortex

The cerebral cortex contain billions of neurons, which contain NOS, an enzyme capable of generating NO. It is not a surprise, therefore, to find endogenous antioxidant such as catalase (CAT), superoxide dismutase (SOD) and glutathione reductase (GRED) in the brain that may be needed to neutralize excess NO and other free radicals. The expression of these enzymes has been shown to be particularly high in the developing brain [49,50]. In fact, it has been reported that these antioxidants may be working in tandem to neutralize H_2_O_2_ in brain culture cells [51].

Here we demonstrated that nociceptin treatment induced a large and significant increase in the expression of CAT in neurons of the cerebral cortex in nociceptin treated rats compared to untreated diabetic controls. This is in contrast to the reduction in the level of expression of CAT in normal rats treated with nociceptin. The reason for this difference is not clear. However, the reason why there is upregulation of CAT in the cerebral cortex of diabetic rats may be due to the fact that the level of CAT in diabetic rat brain is very low compared to that of normal control. Therefore, we might be seeing an upregulation in nociceptin-treated diabetic rats versus a negative feedback in nociceptin-treated normal rats. Indeed, a recent study showed that the use of a herbal plant significantly increased the level of CAT in the cerebral cortex of diabetic mice [52].

The neurons of the cerebral cortex contain SOD, indicating that this enzyme is required for the neutralization of noxious free radicals produced during neuronal metabolism. The expression of SOD was significantly reduced after the onset of diabetes mellitus. However, treatment with nociceptin significantly increased the expression of this endogenous antioxidant in the neurons of the cerebral cortex, especially in diabetic rats, when compared to untreated diabetic rats.

The expression pattern of glutathione reductase (GRED) is similar to that of SOD. Diabetes mellitus caused a significant reduction in the expression of GRED in the neurons of the cerebral cortex compared to normal controls. The administration of nociceptin was associated with a decrease in the expression of GRED in the neurons of the cerebral cortex of normal treated rats and a large, and significant, increase in the cerebral cortex neurons of diabetic rats when compared to their respective controls. A recent study showed that melatonin can increase the level of GRED in the cerebral cortex of diabetic rats [53]. All of these show that GRED is an essential component of the neurons of the cerebral control and could be crucial in protecting neurons from both intrinsic and extrinsic release of reactive oxygen species.

### 4.4. Nociceptin and Endogenous Antioxidants in the CA3 Region of the Hippocampus

The cornu ammonis 3 (CA3) region of the hippocampus, which is located in the dorsal part of the hippocampus, plays an important role in the solidification of memory, be it more recent or those that have happened in the far past [54]. We examined the expression of endogenous antioxidants in this crucial part of the brain to determine whether nociceptin can alter the expression of endogenous antioxidants in this region of the temporal lobe. Nociceptin has been reported to play an important role in several physiological activities processed by the hippocampus, including learning and memory [55]. Since increases in oxidative stress have been reported in many neurological diseases [56], the ability to increase the level of endogenous antioxidants that will neutralize the ROS, generated by oxidative stress, may be key to treating these neurological conditions.

Catalase (CAT) expression was significantly downregulated in diabetic rats compared to controls. This observation corroborates those reported for other regions of the brain [55,56]. Paradoxically, CAT expression was significantly reduced after the administration of nociceptin to either normal or diabetic rats. The reason why the expression level of CAT in the hippocampus was not increased after nociceptin treatment is not clear.

In contrast, superoxide dismutase (SOD) expression was significantly increased in both normal and diabetic rats after nociceptin treatment. This observation shows that SOD may be crucial to the normal functioning of the neurons in this part of the brain. The expression of glutathione reductase (GRED), in this part of the hippocampus, follows the trend of SOD, with an initial decrease after the onset of diabetes. Treatment with nociceptin caused a large increase in the expression of GRED in hippocampal neurons. This indicates that GRED is probably an important tool for the suppression of ROS released by CA3 neurons, and that nociceptin can, indeed, enhance its expression.

### 4.5. Neuronal Nitric Oxide Synthase (nNOS) and cFOS in CA3 Region of Hippocampus

We examined the expression of neuronal nitric oxide synthase (nNOS), first as a marker of neurons, and secondly, whether the expression of this enzyme will decrease after treatment with nociceptin. nNOS is responsible for the production of nitric oxide, a signaling molecule and a maintainer and enhancer of neural plasticity in the nervous system [57]. The administration of nociceptin reduced the expression of nNOS in the hippocampus of normal rats. However, the expression of hippocampal nNOS was significantly enhanced after treatment of diabetic rats with nociceptin. The ability of nociceptin to increase the expression of nNOS in normal rats has been previously reported, a process necessary for the regulation of neuropathic pain in mammals [58]. This shows that nociceptin can, indeed, significantly increase the expression of nNOS, albeit in diabetic rats, in our case. Our observation on the effects of nociceptin on nNOS level in normal rat hippocampus did not corroborate that of Xu et al. [58]. NC treatment has also been shown to mitigate drug-induced disturbances in learning and memory [59].

Since it has been shown that the hippocampus is implicated in the process of learning and memory, we examine the expression of cFOS to determine whether its expression will be altered after the administration of nociceptin. Our results showed that the expression of cFOS was not altered after the administration of nociceptin. The reason for this is not clear. It was expected that, if this region is involved in many neurological processes, then the activation of cFOS in the nuclei of neurons in the part of the brain should be discernible.

## 5. Conclusions

In conclusion, catalase (CAT), superoxide dismutase (SOD), and glutathione reductase (GRED) are widely expressed in the proximal and convoluted tubules of the kidney, hepatocytes and neurons of the cerebral cortex and hippocampus. The administration of nociceptin significantly increased the expression of SOD and GRED in the kidney, hepatocytes of the liver, and neurons of the cerebral cortex and hippocampus, especially in diabetic rats. Nociceptin may be exerting its physiological and neuroprotective actions via increasing the expression of endogenous antioxidants in stressful conditions, such as diabetes mellitus.

### Relevance of the Study and Future Prospectives

This study shows that nociceptin, a neuropeptide involved in many physiological processes, including pain, learning, acquisition of memory, and the release of cytokines and many others, can increase the expression of endogenous antioxidants in many cells, including neurons of the brain. This shows that neurons may be able to defend themselves from free radicals with needed support from neuroglia cells. The pathway by which nociceptin induces the expression of endogenous antioxidant is a topic for future research. The observations of this study may have significant implications in the uses of nociceptin as an enhancer of endogenous antioxidants.

## Figures and Tables

**Table 1:** Details of the primary antibodies used in this study.

#	Antibody	Source	Type	Cat No.	Dilution	Manufacturer
1	Anti-Superoxide dismutase	Rabbit	Polyclonal	ab13498	1:200	Cambridge, MA, USA
2	Anti-Catalase	Rabbit	Polyclonal	ab16731	1:200	Cambridge, MA, USA
3	Anti-Glutathione Reductase	Mouse	Monoclonal	ab16801	1:200	Cambridge, MA, USA
4	Neural NOS	Mouse	Monoclonal	ab610308	1:500	BD Transduction Labs San Jose, CA, USA
5	cFOS	Mouse	Monoclonal	sc-271243	1:100	Santa Cruz Biotechnology, Dallas, TX, USA
6	FITC	Goat	Polyclonal	111-095-003	1:100	Jackson ImmunoResearch Laboratories, Europe Ltd. (Ely, Cambridgeshire, UK)
7	TRITC	Goat	Monoclonal	111-025-003	1:100	Jackson ImmunoResearch Laboratories, Europe Ltd. (Ely, Cambridgeshire, UK)

## Data Availability

All data have been presented and available in the manuscript. Additional information regarding the manuscript will be welcome by the authors.

## Supplementary Materials

The following are available online at https://www.mdpi.com/article/10.3390/biology10070621/s1, Table S1: Characteristics of the animals; Figure S1: Catalase-immunoreactive neurons in the cerebral cortex (thick arrow) and varicose nerves (thin arrow) of diabetic rats treated with nociceptin. Mag. ×400; Figure S2: Superoxidase-immunoreactive neurons in the cerebral cortex (thick arrow) and varicose nerves (thin arrow) of normal rats treated with nociceptin. Mag. ×400; Figure S3: Glutathione reductase-immunoreactive neurons in the cerebral cortex (thick arrow) and varicose nerves (thin arrow) of normal rats treated with nociceptin. Mag. ×400.
