# Supplementary material for: Nociceptin Increases Antioxidant Expression in the Kidney, Liver and Brain of Diabetic Rats
Source: Biology (Basel). 2021 Jul 3;10(7):621. doi: 10.3390/biology10070621 (PMC8301093; doi:10.3390/biology10070621)
Supplement: Supplementary file 1 [file biology-10-00621-s001.zip › biology-1216022-SI.pdf]

biology

Table S1. Characteristics of the animals.

	N	NT	DM	DMT
Weight	242.6 ± 19	251.0 ± 11.98	32.4 ± 16.1	24 ± 17.72
Fasting Blood Glucose	124.8 ± 18	125.4 ± 16	554.4 ± 40.8	497.67 ± 76.2

N=Normal untreated; NT=Normal treated; DM=Diabetes untreated; DMT=Diabeted treated.

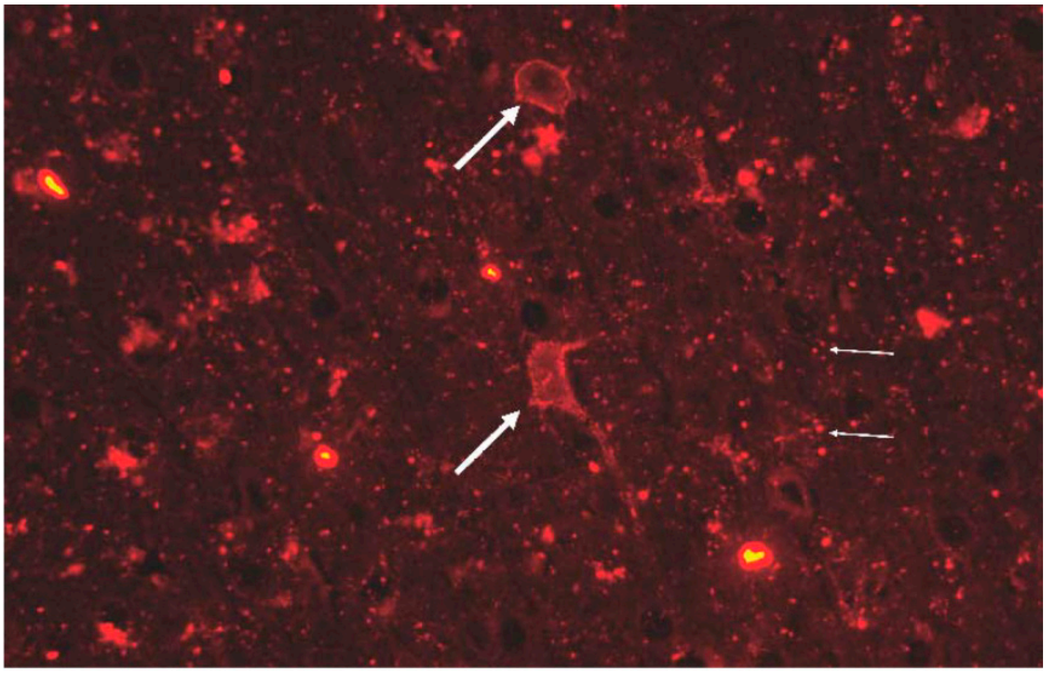

Figure S1. Catalase-immunoreactive neurons in the cerebral cortex (thick arrow) and varicose nerves (thin arrow) of diabetic rats treated with nociceptin. Magnification = ×400.

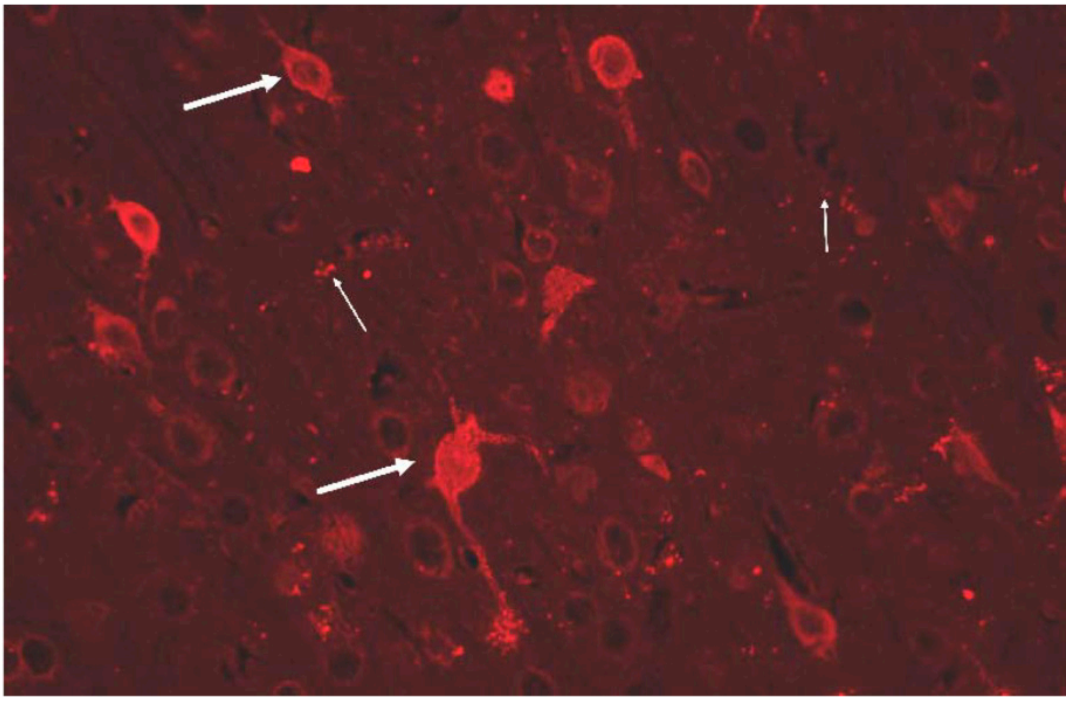

Figure S2. Superoxidase-immunoreactive neurons in the cerebral cortex (thick arrow) and varicose nerves (thin arrow) of normal rats treated with nociceptin. Magnification = ×400.

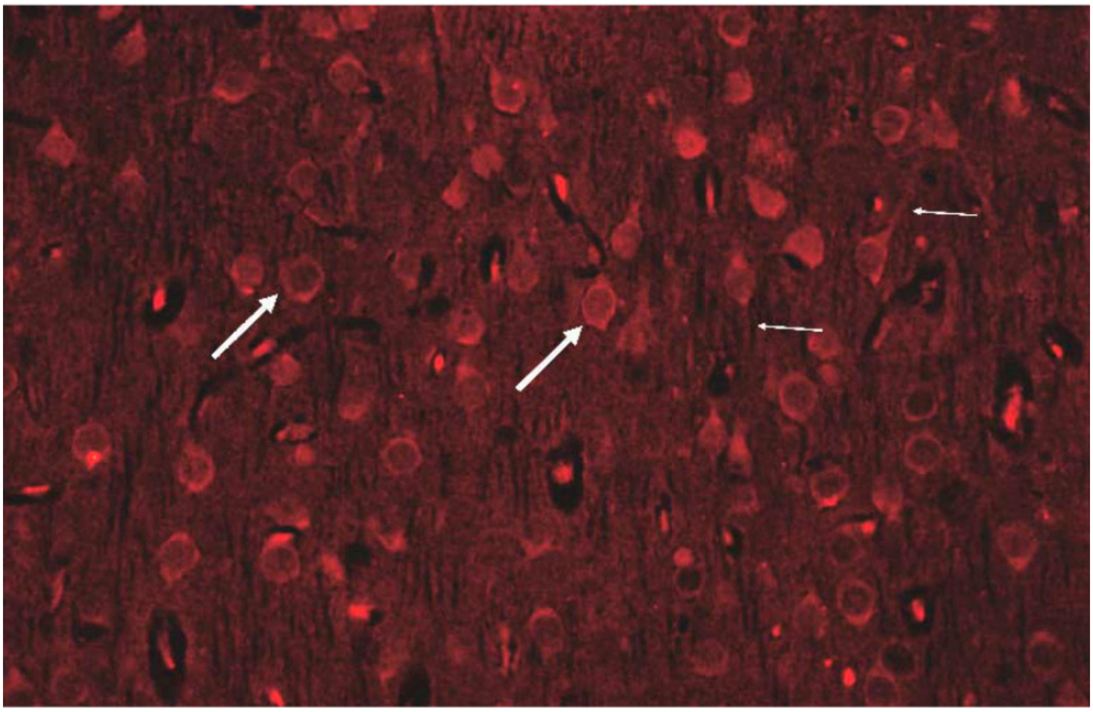

Figure S3. Glutathione reductase-immunoreactive neurons in the cerebral cortex (thick arrow) and dendrites of neurons (thin arrow) of normal rats treated with nociceptin. Magnification = ×400.
